# *In silico* Analysis of Conformational Changes Induced by Mutation of Aromatic Binding Residues: Consequences for Drug Binding in the hERG K+ Channel

**DOI:** 10.1371/journal.pone.0028778

**Published:** 2011-12-15

**Authors:** Kirsten Knape, Tobias Linder, Peter Wolschann, Anton Beyer, Anna Stary-Weinzinger

**Affiliations:** 1 Institute for Theoretical Chemistry, University of Vienna, Vienna, Austria; 2 Department of Pharmacology and Toxicology, University of Vienna, Vienna, Austria; University of Georgia, United States of America

## Abstract

Pharmacological inhibition of cardiac hERG K^+^ channels is associated with increased risk of lethal arrhythmias. Many drugs reduce hERG current by directly binding to the channel, thereby blocking ion conduction. Mutation of two aromatic residues (F656 and Y652) substantially decreases the potency of numerous structurally diverse compounds. Nevertheless, some drugs are only weakly affected by mutation Y652A. In this study we utilize molecular dynamics simulations and docking studies to analyze the different effects of mutation Y652A on a selected number of hERG blockers. MD simulations reveal conformational changes in the binding site induced by mutation Y652A. Loss of π-π-stacking between the two aromatic residues induces a conformational change of the F656 side chain from a cavity facing to cavity lining orientation. Docking studies and MD simulations qualitatively reproduce the diverse experimentally observed modulatory effects of mutation Y652A and provide a new structural interpretation for the sensitivity differences.

## Introduction

HERG (human ether-a-go-go related gene) encodes the pore-forming subunit of the voltage-gated potassium channel I_Kr_ expressed in the heart and in nervous tissue [1]. The channel contributes to modulation of the repolarization phase III of the myocyte action potential [1]–[3]. Disruption of hERG channel function, due to inherited mutations [4], [5], or side effects of drugs, has been linked to long QT syndrome (LQTS) [6], which may lead to serious arrhythmia and sudden cardiac death [7], [8]. This phenomenon is caused by structurally diverse therapeutic compounds including antiarrhythmics, antihistamines, antipsychotics and antibiotics [9]. Several compounds like terfenadine (Seldane®) and cisapride (Propulsid®) had to be withdrawn from the market for this reason. Consequently, there is an intense interest in understanding the molecular and structural mechanisms of hERG channel gating and block. Individual mutations of pore forming residues to alanine revealed amino acids essential for drug binding. Residues T623, S624 and V625, located at the bottom of the pore helix, and residues G648, Y652 and F656, located in S6 segments are important binding determinants for many drugs from diverse chemical classes [1], [10]–[21]. Mutations of Y652 and F656 to alanine resulted in 94-fold and 650-fold block decrease for compound MK-499, respectively [10]. Similar strong effects have been found for many structurally unrelated compounds such as cisapride and terfenadine, suggesting a common binding region within the aqueous inner cavity [22].

**Figure 1:**
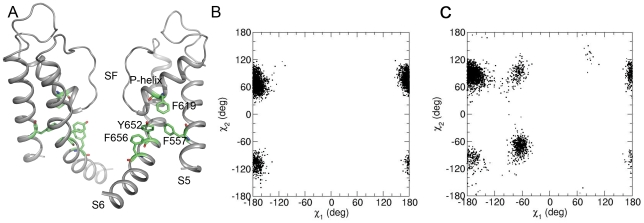
Location and flexibility of putative aromatic binding residues in hERG. (A) π-π-stacking interactions between binding determinants Y652 and F656, located on helix S6 and residues F619 (P-helix) and F557 (S5 helix). Side chains are shown as green sticks (B). χ_1_/χ_2_ plot of Y652 and F656 (C) obtained from 50 ns MD simulations.

Homology models [23]–[27] suggest that high affinity binding determinants Y652 and F656 are arranged in two aromatic rings, facing the inner cavity (Fig. 1). π-π-stacking interactions as well as cation-π-interactions with these residues have been proposed to play a crucial role for block [28]. The importance of the aromatic side chain at position Y652 is further supported by mutational studies, indicating that conservative mutations Y652F and Y652W retain normal sensitivity to high affinity blockers MK-499 and cisapride [10] while non-aromatic substitutions strongly diminish block. In contrast, at position F656 hydrophobicity seems sufficient for high affinity block [16].

The binding mode for blockers such as bepridil, thioridazine or fluvoxamine differs with respect to Y652. These compounds are only partially attenuated by mutation Y652A [22], [28]–[30]. Nevertheless, with the exception of fluvoxamine [28], drugs are strongly attenuated by mutation F656A, suggesting that they bind in the inner cavity [12], [31]. In 2009, Xing et al. [32] found that capsaicin, a pungent irritant occurring in peppers, enhances hERG block upon mutation of Y652A 4-fold, while F656 was suggested to be relatively unimportant for block.

The mechanism by which these drugs interact with hERG channels is largely unknown. Thus, we investigated whether bepridil, thioridazine, propafenone and capsaicin have different binding modes compared to cisapride, dofetilide, E-4031, MK-499, terfenadine or ibutilide. In this study we utilized MD simulations and docking studies to investigate the different role of Y652 on drug binding.

## Results

### Flexibility of putative aromatic binding residues in the hERG cavity

A recently validated homology model of the open hERG pore (model 6 of Stary et al. [26]) was used as starting point for our analyses. Y652 and F656 belong to a cluster of four aromatic residues, which includes F557 located on helix S5 and F619 from the P segment (Figure 1A). The conformational flexibility of these aromatic side chains was analyzed using molecular dynamics simulations. Figure 1B–C shows the distribution of dihedral angles χ_1_ (rotation around Cα–Cβ atoms) and χ_2_ (rotation around Cβ–Cγ atoms) for side chains Y652 and F656 on a 50 ns time scale. Since our sampling protocol involved sampling at 10 ps intervals and each channel contains four homologous domains, each plot contains 20,000 black dots representing the conformations observed in the simulation. Figure 1B illustrates the rigidity of the Y652 side chain on the nanosecond time scale. Variations are observed for the dihedral angle χ_2_ only. The F656 side chain is more mobile, it can adopt various χ_1_ and χ_2_ conformations. The multiple observed conformational states suggest inherited flexibility at position F656 in the open conformation. The side chain of F557, which is not part of the drug binding site, is relatively rigid. The phenyl ring of F619 from the P-helix adopts various χ_1_ and χ_2_ conformations (see Figure S1A–B).

### Conformational changes induced by alanine mutations

**Figure 2:**
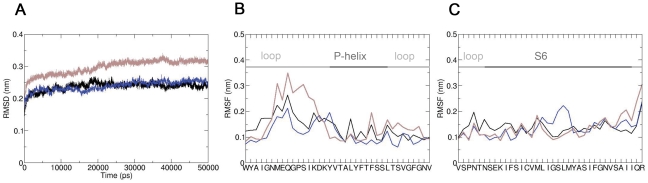
Stability of WT and mutant hERG channels. (A) Backbone RMSD of the Y652A (blue) and the F656A mutant (brown) compared to WT channel (black). (B) Comparison of the root mean square fluctuations (RMSF) for WT and mutant channels. Only the P-helix and connecting loops are shown. (C) RMSF of S6 helix.

The structural effects of mutations Y652A and F656A were examined using MD simulations. First, *in silico* mutants were generated using the mutagenesis tool in PyMOL, followed by energy-minimizations. Repeated simulations on a 50 ns time scale were performed. The stability of the mutant channels, measured as the root mean square deviation (RMSD) as a function of time is shown in Figure 2A. The values for WT and Y652 are in the range of 0.25 nm, the RMSD for the F656A mutant is slightly higher; it reaches 0.3 nm after 50 ns. The increased RMSD is not due to stability differences in S6 helices (Figure 2C) but due to less stable loops connecting S5 and P helix (Figure 2B).

**Figure 3:**
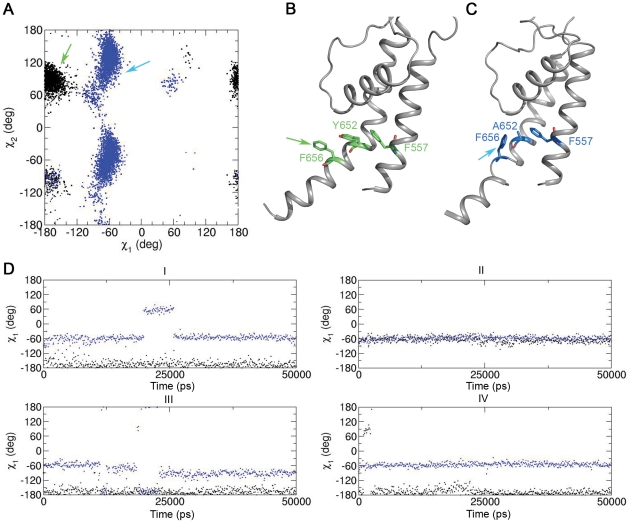
Side chain rearrangements of F656 induced by mutation Y652A. (A) χ_1_/χ_2_ side-chain angles of F656 for WT (black) and Y652A (blue). The green and blue arrows indicate the approximate conformations of the F656 side chains shown in B and C. (B) Representative side-chain conformations of WT and Y652A mutant (C) channel snapshots taken from MD simulations. (D) F656 χ_1_ dihedral angles for WT (black) and Y652A (blue) in all four domains as a function of time.

Replacement of the planar aromatic moiety in position Y652 altered the conformation of residue F656, which was stabilized by parallel displaced π-π-stacking interactions in WT. Calculations by Tsuzuki et al. [33], indicate that the energy contribution for this type of aromatic-aromatic interactions is in the range of −1.48 kcal/mol. Due to the loss of these interactions in the Y652A mutant the side chain of F656 rotated away from the pore axis allowing edge to edge shaped π-π-stacking interactions with F557 from the neighboring S5 segment (see Figure 3A–C). The interaction energies of edge to edge π-π-stacking are approximately 1 kcal/mol stronger than parallel displaced π-π-stacking (−2.48 kcal/mol vs. −1.48 kcal/mol [33]). Figure 3D compares the χ_1_ angle of side chain F656 in WT and Y652A mutant channels as a function of time. The χ_1_ angle is predominantly in the range of −180° to −60° in WT (trans orientation). In the mutant channel this value is changed to −60° to 60°. The more favorable edge to edge stacking energy might explain why the F656 side chain adopted a gauche(−) conformation in 80% of the simulations. Gauche(+) and trans conformations were rarely observed (results for the rerun are shown in Figure S3).

MD simulations on the F656A mutant did not reveal significant conformational changes of aromatic residues compared to WT (see Figure S1A–C). Therefore, this mutant was not analyzed further.

### Docking studies on WT and Y652A mutant channels

**Figure 4:**
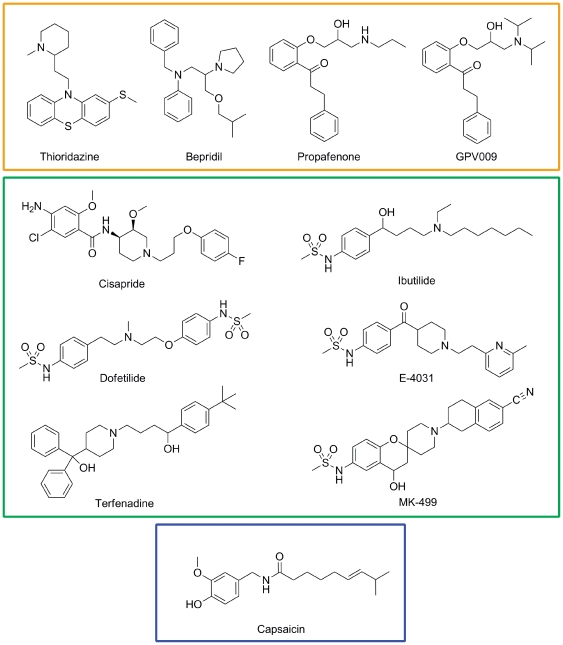
Structures of hERG blockers examined in this study. Drugs are clustered into three groups: group 1 (orange frame) includes blockers which are relatively insensitive to mutation Y652A^22,28–30^, group 2 (green frame) shows Y652 sensitive drugs^10,18,22,31^ and group 3 (blue frame) shows capsaicin whose affinity is increased by mutation Y652A^32^.

**Figure 5:**
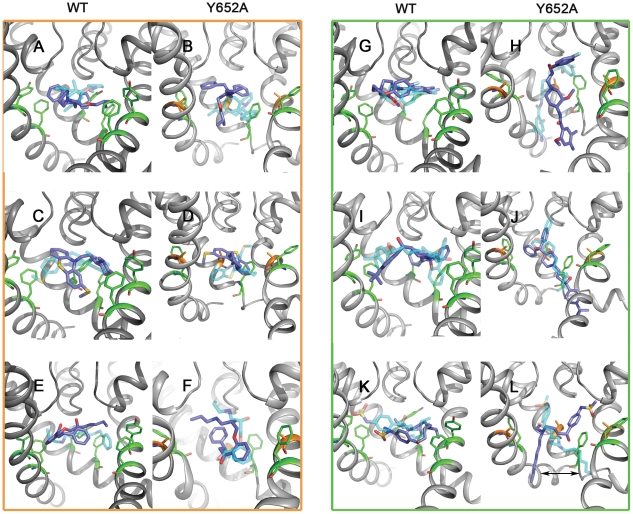
Docking (cyan transparent sticks) and MD poses. at the end of the simulation (blue sticks) of bepridil (AB), thioridazine (CD), propafenone (EF), cisapride (GH), terfenadine (IJ) and ibutilide (KL) in WT and Y652A (from left to right). Y652 and F656 are shown as green lines; A652 is shown as orange lines. The arrow displays the movement of the heptyl chain of ibutilide.

We next analyzed the effects of the Y652A mutant induced side chain orientation of residue F656 on drug block. Eleven drugs (Figure 4 and Figure 5) were docked into 20 WT and 20 Y652A snapshots (every 5 ns from two independent runs) derived from 50 ns MD simulations. For each blocker, the ten most frequent occurring docking poses of each drug (n = 100) were analyzed with respect to aromatic ring stacking and/or hydrophobic interactions with binding residues Y652 and F656. Table 1 summarizes these interactions and lists the number of t-shaped (t), edge-to-edge (e) and parallel π-π-stacking (p) interactions. Gold Chemscores (Gold.Chemscore.DG) are listed in Table 2.

**Table 1:** Aromatic ring stacking and hydrophobic interactions (HIA) between Y652 and F656 side chains and hERG antagonists in WT and Y652A mutant channels.

Compound	WT			Y652A		
	Y652	F656	∑ HIA	A652	F656	∑ HIA
**Thioridazine**	-	3 (t,p,e)	3	-	3 (t,p,e)	3
**Bepridil**	1 (p)	1 (e)	2	-	2 (e)	2
**Propafenone**	2 (t,e)	2 (t,e)	4	-	2 (e)	2
**GPV0009**	3 (t,2e)	1 (e)	4	-	1 (e)	1
**Capsaicin**	-	1 (p)	1	-	1 (e)	1
**Cisapride**	2 (p,e)	2 (t,p)	4	-	-	0
**Dofetilide**	2 (t,e)	2 (p,e)	4	-	1 (e)	1
**E-4031**	3 (t,2e)	2 (e)	5	-	2 (p,e)	2
**Ibutilide**	3 (e)	-	3	-	1 (p)	1
**MK-499**	1 (e)	1 (e)	2	-	-	0
**Terfenadine**	3 (t,2e)	2 (t,e)	5	-	1 (e)	1

(t = T-shaped stacking, p = parallel π-π-stacking, e = edge-to-edge interactions).

**Table 2:** Free energies of binding calculated by Chemscore (ΔG^bind^ kJ/mol) for WT and Y652A.

Drug	ChemscoreWT	ChemscoreY652A	DifferenceWT vs Y652A
**Thioridazine**	−30.67	−27.83	−2.84
**Bepridil**	−32.92	−31.23	−1.69
**Propafenone**	−32.88	−29.75	−3.13
**GPV0009**	−35.51	−31.43	−4.08
**Capsaicin**	−31.34	−32.85	1.51
**Cisapride**	−30.86	−22.96	−7.90
**Dofetilide**	−30.00	−20.67	−9.33
**E-4031**	−35.59	−22.82	−12.77
**Ibutilide**	−33.69	−21.52	−12.17
**MK-499**	−30.18	−23.19	−6.99
**Terfenadine**	−35.21	−30.51	−4.70

Drugs can be divided into three groups according to their binding behavior. For drugs that have been shown to be only partially attenuated by a tyrosine to alanine mutation in position 652 [22], [28]–[30], no or slight changes in binding behavior compared to WT were observed (Table 1 and Figure 5). The binding mode for thioridazine was identical in WT and Y652A. Three aromatic interactions were predicted in both cases (Figure 5). Docking studies with bepridil suggested that the total number of aromatic interactions remained constant in the mutant channel. However, in the WT channel this drug formed one parallel π-π-stacking interaction with Y652 and one edge-to-edge interaction, while in the Y652A mutant channel, two edge-to-edge interactions with F656 were predicted (Figure 5). The number of aromatic interactions for propafenone and GPV009 did not change in Y652A. The only modification observed was a change of one t-shaped to an edge-to-edge stacking interaction with propafenone.

In agreement with experimental data, cisapride, dofetilide, E-4031, ibutilide, MK-499 and terfenadine were predicted to strongly interact with aromatic side chains Y652 and F656. While several favorable aromatic interactions to both aromatic side chains were predicted for cisapride and MK-499 in the WT channel, docking studies performed with the Y652A mutant channel indicated complete loss of aromatic interactions. All other drugs in this group had drastically reduced aromatic and hydrophobic interactions with F656 in the Y652A mutant channel. For example, in the WT channel terfenadine was predicted to interact with Y652 side chains from three domains and two F656 residues. In the mutant channel only one edge-to-edge interaction with the F656 side chain remained (see Figure 5 and Figure S2).

Important changes between the Y652A sensitive and Y652A insensitive drug groups were also observed considering the conformation of the drugs. Docking results suggest that thioridazine, bepridil, propafenone and GPV009 fold mostly into U-shaped conformations, while extended conformations parallel to the pore axis were not observed in either WT or Y652A mutant channel. In contrast, most drugs that are highly sensitive to mutation Y652A change their conformation from U-shaped in the WT channel to a stretched conformation longitudinal to the channel axis (Figure 5).

**Figure 6:**
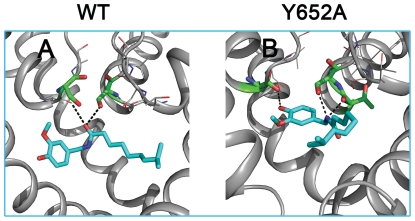
Interactions of capsaicin with the selectivity filter in WT (A) and Y652A mutant (B). Selectivity filter residues involved in capsaicin binding are shown as green sticks; residues of the TSV motif not interacting with capsaicin are shown as grey lines. Hydrogen bonds are depicted as black dots.

Capsaicin possibly belongs to a third class of drugs, which is affected by the Y652A mutation in a different way. To the best of our knowledge, it is currently the only known drug that shows increased affinity for the Y652A mutant. Gold predicted one aromatic interaction (parallel or edge-to-edge) for WT and Y652A, respectively. Docking suggests that the number of hydrogen bonds with selectivity filter residues T623 and S624 increased in the mutant channel (two H-bonds to T623 and one H-bond to S624). In the WT channel only two hydrogen bonds between capsaicin and S624 were predicted (Figure 6).

### MD simulations support different drug binding modes in WT and Y652A channels

To further support our hypothesis, 10 ns MD simulations on all docked poses shown in Figure 5 and Figure S2 were performed. Generally, the docked binding poses are stable on the nanosecond time scale. The Y652A insensitive compounds bepridil, thioridazine, propafenone, and GPV009 retain their compact binding mode in the channel pore in WT and Y652A mutant channels (Figure 5A–F, Figure S2A–B). For the drugs which are sensitive to mutation Y652A, simulations strongly support the suggested drug rearrangement from the horizontal binding mode in WT to a stretched conformation along the channel axis in the mutants (Figure 5G–L, Figure S2C–H). This provides a possible explanation for the experimentally observed affinity loss. Only E-4031 does not remain stable in the Y652A mutant (Figure 5J).

Additionally, MD simulations reveal which functional groups of the compounds are flexible. For example, while the basic scaffold of the propafenone molecule (acylphenyloxypropanolamine) remains rather rigid, the side chain adopts various conformations. The movies S1, S2, S3, S4, S5, S6, S7, S8, S9, and S10 show the behavior of all ten drugs in WT and mutant channels during the 10 ns MD simulation runs.

Surprisingly, the conformational flexibility of the Y652 and F656 side chains is not influenced when drugs reside in the cavity (for examples see Figure S6). In contrast, conformational changes of the aromatic side chains sometimes induce changes in drug orientation (for example see behavior of E-4031 in the movie S5).

## Discussion

Direct block of hERG channels by structurally diverse drugs is mediated by aromatic side chains Y652 and F656 (see Figure 1 for location of residues) [17]. Mutation of either residue to alanine dramatically reduces drug potency, implying a direct interaction with these residues. In agreement with this hypothesis, various drug docking studies predict binding modes, favoring π-π-stacking interactions with Y652 and F656.

More recently, compounds have been identified, which are insensitive to mutation Y652A, while displaying greatly reduced affinity for the F656A mutant [22], [28], [29]. The lack of sensitivity of these molecules could simply result from binding “less deeply” in the cavity, possibly below the position of Y652. Alternatively, replacement of Y652 might induce allosteric effects on drug binding rather than directly disrupting binding. For the reasons discussed in detail below, we favor the second hypothesis.

Our MD simulations suggest that deletion of the aromatic side chain in position Y652 induces allosteric changes in the drug binding site, with important consequences for drug binding. Specifically, loss of π-π-stacking interactions induce a conformational change of the F656 side chain from a cavity facing orientation (χ_1_ values in the range of −180°) to a cavity lining conformation (χ_1_ values in the range of −60°) (compare Figure 1A–C and 3A–C). This conformation is stabilized by energetically favorable edge-to-edge stacking interactions with the F557 aryl ring, located on helix S5. Docking studies comparing the binding modes of 11 hERG blockers revealed different behavior for rigid compact molecules versus compounds with more extended geometries. Only the first class of drugs could still favorably interact with the reoriented F656 residues from several subunits in the Y652A mutant (Figure 5, Table 1 and Figure S2). These results correlate well with experimental Y652A sensitivities and are in agreement with a ligand based hypothesis by Stansfeld et al. [34], [35] derived from a study of 20 LQT compounds with varying Y652A sensitivities. Besides, the importance of the orientation of the Y652 and F656 side chains for high affinity block has been elegantly demonstrated by Chen et al. [36] Their study showed that the decreased drug affinity of non-inactivating hERG mutant channels is not caused by inactivation per se but by inactivation gating-associated reorientation of residues located in the S6 domain.

It has been reported by Zachariae et al. [37] that longer molecules bind in a perpendicular orientation to the channel axis and therefore may interact with all four domains of the channel. In our WT drug docking studies we observe the same perpendicular positioning. In the Y652A mutant, the orientation of extended compounds, sensitive to mutation of Y652 is changed to a stretched conformation parallel to the channel axis. These drug reorientations in the Y652A mutant are further supported by a total of 200 ns (10 and for WT and mutants, respectively) MD simulations.

In contrast to an interesting study by Huang et al. [38], who observed an induced fit of a toxin binding to the extracellular side of the selectivity filter in a shaker K^+^ channel, we did not see conformational adaptions of Y652 or F656 upon drug binding to the hERG inner cavity (Figure S5). This suggests different drug receptor interactions for different binding sites, which might be in part explained by the different nature of interactions (mainly electrostatic versus mainly aromatic/hydrophobic). Interestingly, conformational changes of the aromatic side chains sometimes even induce changes in drug orientation (see movies S1, S2, S3, S4, S5, S6, S7, S8, S9, and S10).

In a recent review by Zhou et al. [39], it was pointed out that aromatic side chains are predestinated to serve as channel gates, preventing ion flow in the closed conformation. Detailed inspection of our recently published closed hERG homology model [40] indeed reveals an optimal arrangement of the F656 side chains to prevent ion flow. In this study, the side chain reorientations in the Y652A mutant most likely do not influence ion conductance in the open state, however we cannot exclude gating-associated reorientations. It was beyond the scope of the current study to analyze mutation induced effects in the closed channel state. Nevertheless, future studies might provide an answer to this important question.

To further support the allosteric side chain rearrangement hypothesis, we introduced a phenylalanine at position Y652, which was shown to restore WT-like binding behavior for the high affinity compound MK-499 [20]. MD simulations show that in the Y652F mutant the aromatic side chains of F557, F619 and F656 behave similar as in the WT channel (see Figure S4A–D). In agreement with experimental data, we find similar docking poses for WT and Y652F channels with compound MK-499.

Allosteric effects on drug block are also used to explain the effects of several inactivation deficient mutants on hERG block. For example, inactivation deficient mutants N588K and S620T exhibit reduced affinity for dofetilide and other high affinity blockers [41]. However, none of these residues is assumed to directly interact with these compounds, since both are located distantly from the binding site.

In conclusion, MD simulations of WT and Y652A mutant channels in combination with drug docking provide a new structural interpretation for the diverse modulatory effects of residue Y652 on different hERG blockers ranging from strong affinity decrease (e.g. cisapride) upon mutation to affinity increase in the case of capsaicin. The results provide a starting point for future investigations focusing on further residues of the aromatic cluster in the hERG binding site. For example, studies on mutants of F557 and F619 will provide a better understanding of the still poorly described mechanisms underlying hERG block.

## Materials and Methods

### Molecular dynamics simulations

MD simulations were performed with Gromacs v.4.5.4. [42] Two independent simulation setups using either the OPLS-all-atom force field [43] or the amber99sb force field [44] were used to analyze the dynamics of hERG WT and mutant channels. Mutants Y652A, Y652F and F656A were generated using the mutagenesis tool in PyMOL 0.99 [45] In the OPLS setup, hERG WT and mutant channels were embedded in an equilibrated simulation box of 241 palmitoyloleoylphosphatidylcholine (POPC) lipids. The channels were inserted into the membrane as described previously [26]. K^+^ ions were placed in the channel at K^+^ sites S0, S2, and S4, with waters placed at S1 and S3 of the selectivity filter [46]. Cl^−^ ions were added randomly within the solvent to neutralize the system. Lipid parameters were taken from Berger et al. [47]. The solvent was described by the TIP4P water model [48]. Electrostatic interactions were calculated explicitly at a distance <1 nm and long-range electrostatic interactions were calculated at every step by particle-mesh Ewald summation [49]. Lennard–Jones interactions were calculated with a cutoff of 1 nm. All bonds were constrained by using the LINCS algorithm [50], allowing for an integration time step of 2 fs. The Nose-Hoover thermostat was used to keep simulation temperature constant by weakly (*τ* = 0.1 ps) coupling the lipids, protein and solvent (water+counter-ions) separately to a temperature bath of 300 K. The pressure was kept constant by weakly coupling the system to a pressure bath of 1 bar using a semi-isotropic Parrinello-Rahman barostat algorithm with a coupling constant of 1 ps. Prior to simulations, 1000 conjugate gradient energy-minimization steps were performed, followed by 2 ns of restrained MD in which the protein atoms were restrained with a force constant of 1000 kJ/mol^−1^ nm^−2^ to their initial position. Ions, lipids and solvent were allowed to move freely during equilibration. The systems were then subjected to 50 ns (15 ns Y652F) of unrestrained MD, during which coordinates were saved every 10 ps for analysis. Residues at the N- and C-termini were considered as uncharged, as neither lie at the actual termini of the complete channel. In the amber99sb setup hERG WT and mutant channels were embedded in an equilibrated membrane consisting of 280 dioleolylphosphatidylcholine (DOPC) lipids. Lipid parameters were taken from Siu, et al. [51] and the TIP3P water model [48] was utilized. All further parameters and steps were carried out as described above. Drug topologies were generated using antechamber, which is part of the Amber 11 program package [52]. Charges were taken from Gaussian runs described in the docking section below. After energy-minimization (1000 conjugate gradient energy-minimization steps), unrestrained 10 ns MD simulations for each compound were carried out for WT and Y652A (200 ns in total) at 310 K.

### Drug docking

Coordinates of the drugs were generated with Gaussview 5 [53] and the geometry optimized with HF/3-21G implemented in Gaussian09 [53]. For thioridazine, propafenone, GPV009, terfenadine, MK-499 and ibutilide (R)- and (S)-conformations were docked. As no differences could be observed between both enantiomers, only the (R)-conformation was used for further analysis. Docking was performed with the program Gold 4.0.1 [54] using the Gold and Chemscore scoring functions. The coordinates of the geometric center calculated among the Y652 and F656 residues were taken as binding site origin. The binding site radius was set equal to 10 Å. 100,000 operations of the GOLD genetic algorithm were used to dock the selected compounds into the WT and mutant channels. Snapshots after 8, 10, 15, 20, 25, 30, 35, 40, 45 and 50 ns were taken from our 50 ns or 15 ns (Y652F) MD trajectories. The best ranked 100 poses of each docking run were used for visual analysis of binding. From the ten most occurring positions the numbers of aromatic interactions were averaged.

## Supporting Information

Figure S1 **χ_1_/χ_2_ plots for F557 (A), F619 (B) and Y652 (C) in hERG WT channel (black) and F656A (brown).**
(TIF)

Figure S2 **GPV009 (AB), MK-499 (CD), E-4031 (EF) and dofetilide (GH) in WT and Y652A (from left to right).** Cyan transparent sticks show the docking pose and blue sticks the MD pose and the end of the simulation. The black arrow indicates the moving direction of E-4031 (the dynamical movement of the drug can be observed in the attached movie).
(TIF)

Figure S3 **MD simulation rerun (50 ns) for Y652A mutant. RMSD plot (A), χ_1_/χ_2_ plot for F656 (B) and the F656 χ_1_ dihedral angles in all four domains as a function of time (C) show no significant deviation from the original run.**
(TIF)

Figure S4 **χ_1_/χ_2_ plots for F557 (A), F619 (B), Y/F652 (C) and F656 (D) in hERG WT channel (black) and Y652F (green).** The 50 ns MD simulation shows that the flexibility of the aromatic side chains in the mutant is comparable to the WT channel.
(TIF)

Figure S5 **Y652 (A) and F656 (B) χ_1_ dihedral angles as a function of time for WT channel without ligand (black) and with bound bepridil (blue) and dofetilide (red).** C shows the χ_1_ dihedral angles of F656 in the Y652A mutant as a function of time.
(TIFF)

Movie S1 **Behavior of docked drug cisapride in a 10 ns MD simulation.** In the first part of the movie, the drug behavior in the WT is shown, followed by the Y652A mutant simulation. Y652 and F656 are shown as green lines; A652 is shown as orange lines.
(WMV)

Movie S2 **Behavior of docked drug terfenadine in a 10 ns MD simulation.** In the first part of the movie, the drug behavior in the WT is shown, followed by the Y652A mutant simulation. Y652 and F656 are shown as green lines; A652 is shown as orange lines.
(WMV)

Movie S3 **Behavior of docked drug ibutilide in a 10 ns MD simulation.** In the first part of the movie, the drug behavior in the WT is shown, followed by the Y652A mutant simulation. Y652 and F656 are shown as green lines; A652 is shown as orange lines.
(WMV)

Movie S4 **Behavior of docked drug MK-499 in a 10 ns MD simulation.** In the first part of the movie, the drug behavior in the WT is shown, followed by the Y652A mutant simulation. Y652 and F656 are shown as green lines; A652 is shown as orange lines.
(WMV)

Movie S5 **Behavior of docked drug E-4031 in a 10 ns MD simulation.** In the first part of the movie, the drug behavior in the WT is shown, followed by the Y652A mutant simulation. Y652 and F656 are shown as green lines; A652 is shown as orange lines.
(WMV)

Movie S6 **Behavior of docked drug dofetilide in a 10 ns MD simulation.** In the first part of the movie, the drug behavior in the WT is shown, followed by the Y652A mutant simulation. Y652 and F656 are shown as green lines; A652 is shown as orange lines.
(WMV)

Movie S7 **Behavior of docked drug bepridil in a 10 ns MD simulation.** In the first part of the movie, the drug behavior in the WT is shown, followed by the Y652A mutant simulation. Y652 and F656 are shown as green lines; A652 is shown as orange lines.
(WMV)

Movie S8 **Behavior of docked drug thioridazine in a 10 ns MD simulation.** In the first part of the movie, the drug behavior in the WT is shown, followed by the Y652A mutant simulation. Y652 and F656 are shown as green lines; A652 is shown as orange lines.
(WMV)

Movie S9 **Behavior of docked drug propafenone in a 10 ns MD simulation.** In the first part of the movie, the drug behavior in the WT is shown, followed by the Y652A mutant simulation. Y652 and F656 are shown as green lines; A652 is shown as orange lines.
(WMV)

Movie S10 **Behavior of docked drug GPV0009 in a 10 ns MD simulation.** In the first part of the movie, the drug behavior in the WT is shown, followed by the Y652A mutant simulation. Y652 and F656 are shown as green lines; A652 is shown as orange lines.
(WMV)
