# Supplementary figures and images for: In silico Analysis of Conformational Changes Induced by Mutation of Aromatic Binding Residues: Consequences for Drug Binding in the hERG K+ Channel
Source: PLoS One. 2011 Dec 15;6(12):e28778. doi: 10.1371/journal.pone.0028778 (PMC3240635; doi:10.1371/journal.pone.0028778)

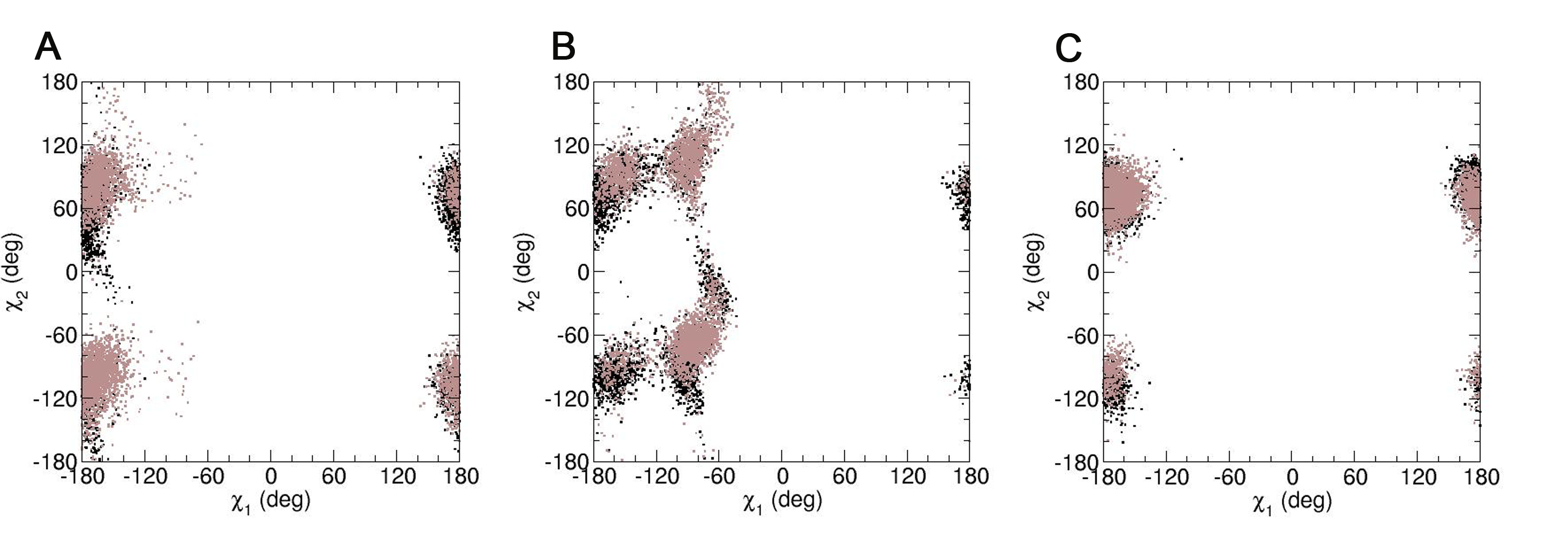

Supplement: Figure S1 — χ1/χ2 plots for F557 (A), F619 (B) and Y652 (C) in hERG WT channel (black) and F656A (brown). (TIF) [file pone.0028778.s001.tif]

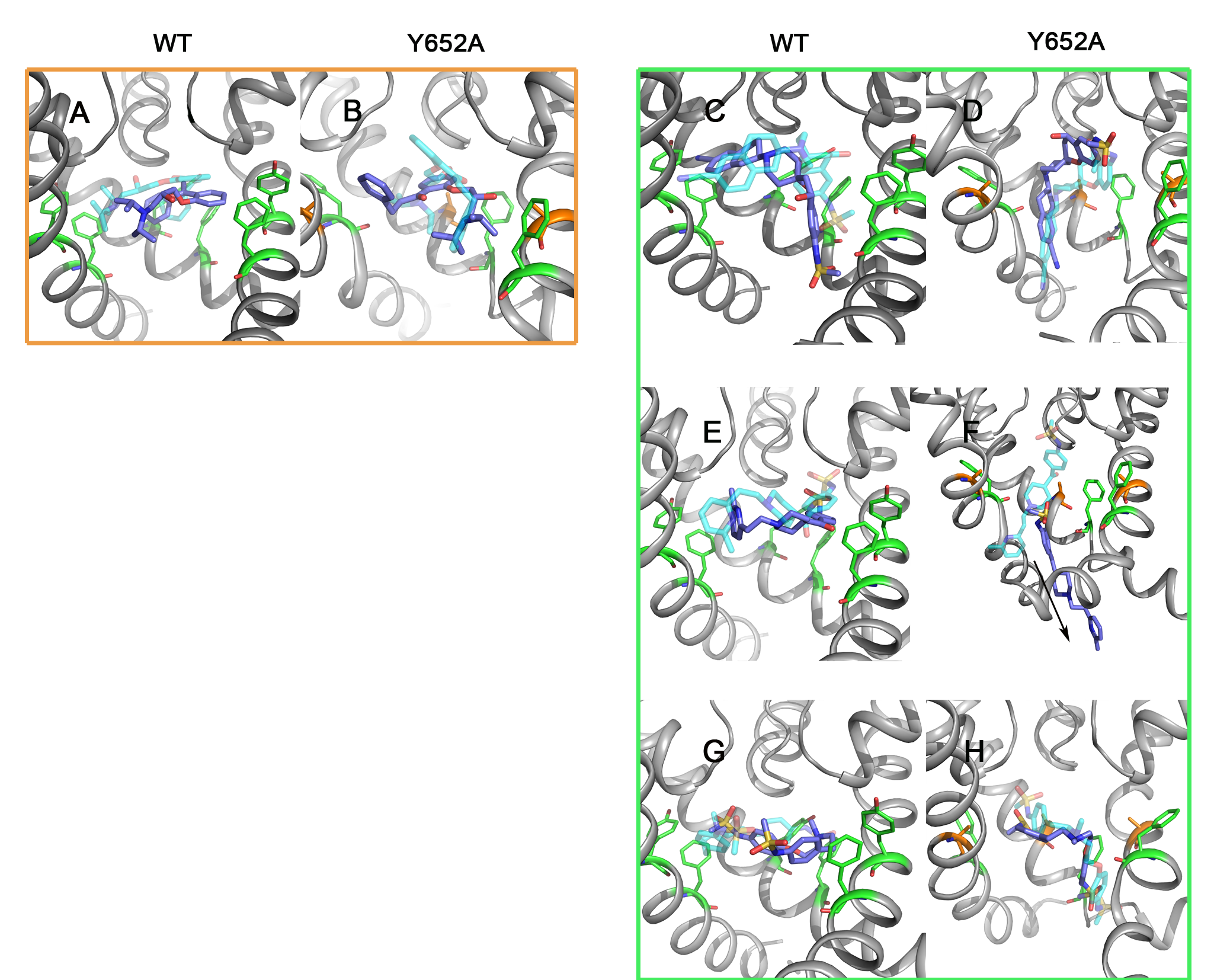

Supplement: Figure S2 — GPV009 (AB), MK-499 (CD), E-4031 (EF) and dofetilide (GH) in WT and Y652A (from left to right). Cyan transparent sticks show the docking pose and blue sticks the MD pose and the end of the simulation. The black arrow indicates the moving direction of E-4031 (the dynamical movement of the drug can be observed in the attached movie). (TIF) [file pone.0028778.s002.tif]

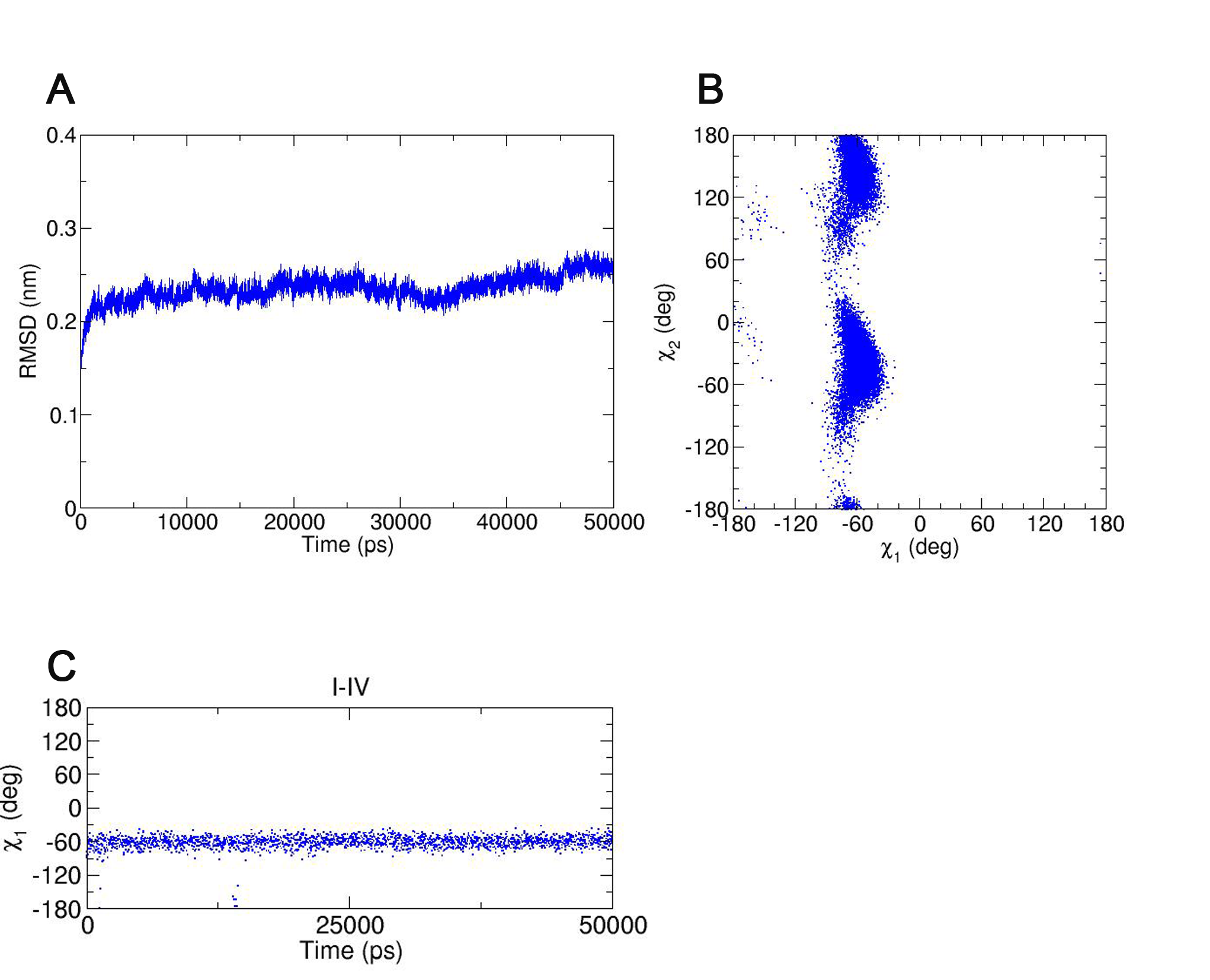

Supplement: Figure S3 — MD simulation rerun (50 ns) for Y652A mutant. RMSD plot (A), χ1/χ2 plot for F656 (B) and the F656 χ1 dihedral angles in all four domains as a function of time (C) show no significant deviation from the original run. (TIF) [file pone.0028778.s003.tif]

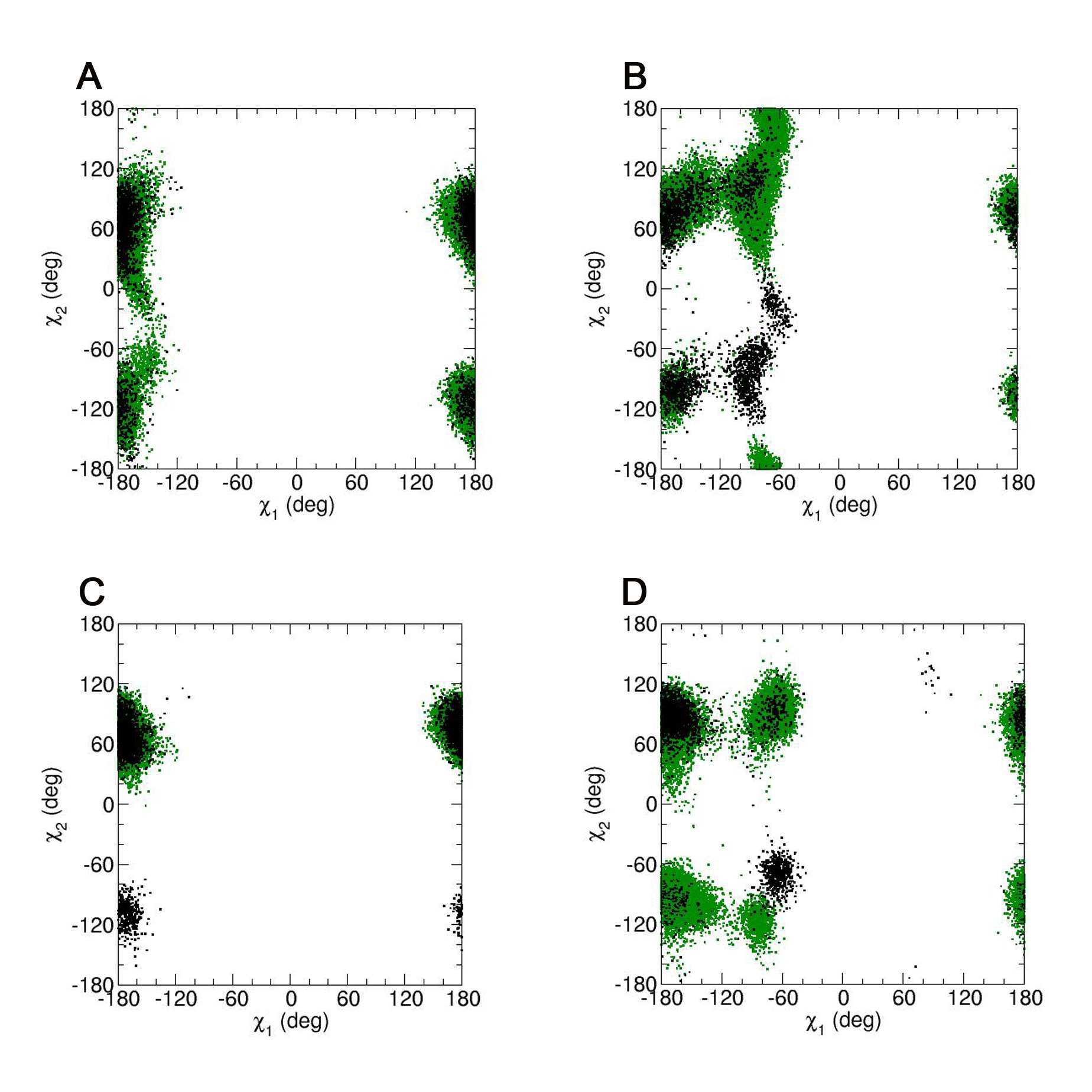

Supplement: Figure S4 — χ1/χ2 plots for F557 (A), F619 (B), Y/F652 (C) and F656 (D) in hERG WT channel (black) and Y652F (green). The 50 ns MD simulation shows that the flexibility of the aromatic side chains in the mutant is comparable to the WT channel. (TIF) [file pone.0028778.s004.tif]

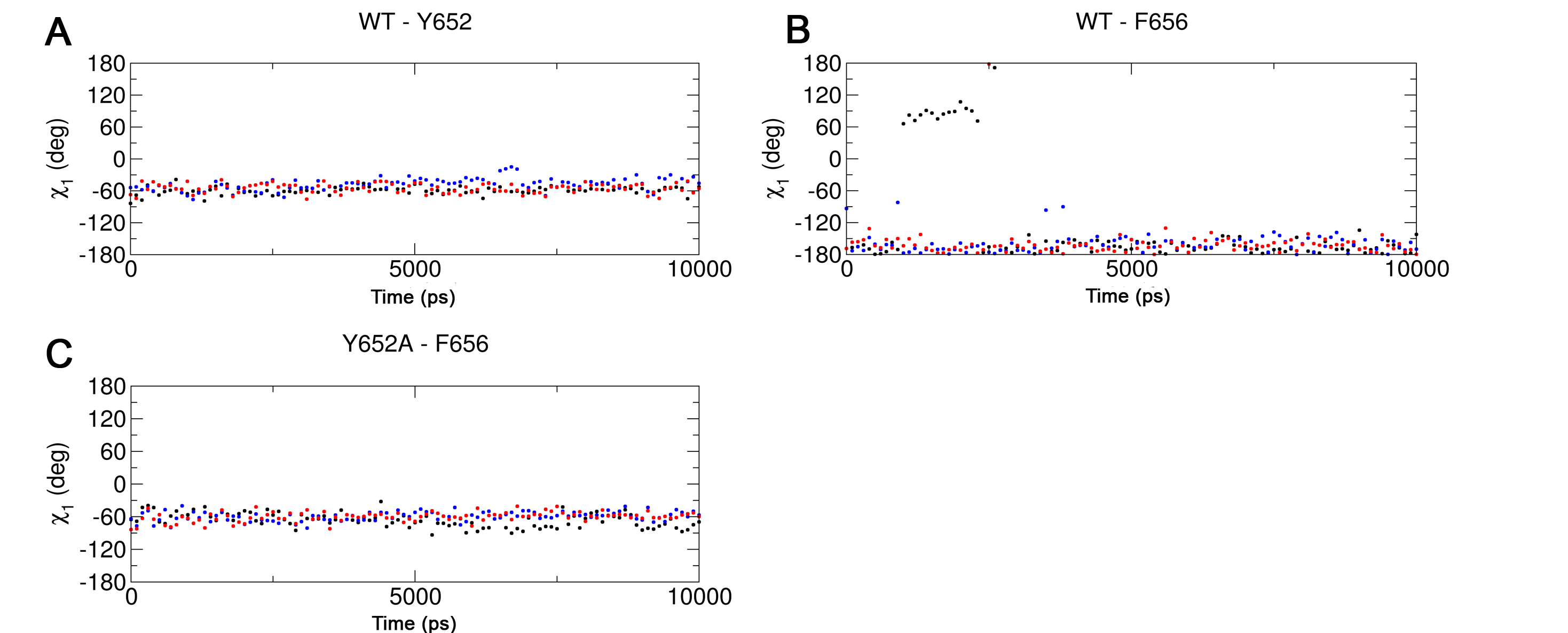

Supplement: Figure S5 — Y652 (A) and F656 (B) χ1 dihedral angles as a function of time for WT channel without ligand (black) and with bound bepridil (blue) and dofetilide (red). C shows the χ1 dihedral angles of F656 in the Y652A mutant as a function of time. (TIFF) [file pone.0028778.s005.tif]
